# Dynamic Profile of Prognostic Hematologic Indicators in Patient Under Intensive Care for COVID-19 Disease: A One-Year Study at a Tertiary Care Centre in South India

**DOI:** 10.7759/cureus.19585

**Published:** 2021-11-15

**Authors:** Jayalakshmi Balasubramanian, Febe Renjitha Suman, Immanuel Ratan Stephen, Sri Gayathri Shanmugam, Rajkumar Mani, Bhargavi Mathan, Lakshmi P

**Affiliations:** 1 Pathology, Sri Ramachandra Institute of Higher Education and Research, Chennai, IND; 2 Pathology and Laboratory Medicine, Sri Ramachandra Institute of Higher Education and Research, Chennai, IND; 3 Internal Medicine, Sri Ramachandra Institute of Higher Education and Research, Chennai, IND

**Keywords:** neutrophilia, medical intensive care unit (micu), platelet to lymphocyte ratio (plr), covid 19, neutrophil to lymphocyte ratio (nlr)

## Abstract

Introduction

Viral pneumonia caused by severe acute respiratory syndrome coronavirus 2 (SARS COV-2) releases cytokines which result in neutrophils migration to the bloodstream and cytotoxic effect on lymphocytes. The ongoing pathology is reflected in the derangement of blood cells and the variations and calculations based on them that help in assessing the severity of the disease and prognosis.

Aim

This study aimed to compare the differences in the dynamic changes of the blood cells among survivors and non-survivors of COVID-19 disease so that cut-offs can be arrived at to aid triage at the intensive care unit (ICU) and to predict mortality.

Material and methods

A one-year study was conducted on patients hospitalized in the ICU. The demography and laboratory values of neutrophils and lymphocytes in percentages and absolute values, and platelet count in numbers were retrieved for eight consecutive values. Neutrophil to lymphocyte ratio (NLR) and platelet to lymphocyte ratio (PLR) was calculated from absolute counts. Statistical analysis was done using the Chi-Square test and Mann-Whitney test and a P-value of <0.05 is considered significant. The comparison was done between survivors and non-survivors.

Result

Among the 3142 patients admitted for COVID-19 disease, 7.6% required ICU care of whom 65.5% survived and 35.5% succumbed to the illness. Survivors were younger and comparable between both sexes. Though both groups had an ascending trend of neutrophils, lymphocytes, NLR, and PLR, the baseline characteristics were significantly lower in those who survived on a day-to-day basis. Neutrophilia above 80%, NLR 7.96, PLR 200 predicted the need for admission in ICU. Neutrophilia of 87% and lymphopenia of 10% were associated with adverse outcomes (mortality). Mortality can be predicted when neutrophil rises above 93% or lymphocytes fall below 5.2%. An initial NLR of 7.96 and PLR of 160 as well as peak NLR of 12.29 and peak PLR 400 predict mortality.

Conclusion

Serial blood counts are essential for hospitalized patients with COVID-19 for early triaging, and to assess severity and prognosis. The NLR of 6.7 and PLR of 160 require intensive care. The dynamic increase of NLR and PLR show worsening of the disease process and NLR of 40.95 and PLR of 400 predict mortality.

## Introduction

The World Health Organisation declared COVID-19 a global pandemic on March 11, 2020 [1]. The outbreak of viral pneumonia caused by severe acute respiratory syndrome coronavirus 2 (SARS COV-2) in Wuhan, China in December 2019, has since taken a heavy toll on India with 3,02,79,331 cases and 3,96,730 deaths [2]. With ongoing mutations of the virus, heterogeneity in infectivity, and clinical presentation, triaging patients by assessing the risk at the time of admission and counselling the patient and the family members have become of utmost importance. Many biomarkers have been evaluated at different institutions among patients with COVID-19 disease. We aimed to study the WBCs and platelets, and the neutrophil-lymphocyte ratio (NLR) and the platelet lymphocyte ratio (PLR) on admission as well as the trend in the course of the disease in patients with severe disease who required management in the ICU. We also attempted to derive cut-off values to predict mortality.

## Materials and methods

A retrospective observational study was conducted for one year. The institutional ethics committee and the Indian Council of medical research approved the study (approval no.CSP-MED/20/SEP/61/75). The study population included adult patients (age > 18 years) with COVID-19 disease admitted to the ICU of our tertiary care centre attached to the super speciality medical college. The patients had positive reverse transcriptase (RT-PCR) report for SARS-COV-2 and typical radiological diagnosis. The study population was divided into two groups based on the outcomes as those who survived and were discharged, and those who died. Demographics of the patient and the outcome were retrieved from the hospital information system and with the help of the Department of Medicine. Laboratory details, namely differential percentage of neutrophils and lymphocytes as well as platelet counts, were retrieved from the laboratory information system. The absolute counts were retrieved from the fully automated hematology analyser post which, the NLR and PLR were calculated. The laboratory details on Day 1 of admission as well as eight consecutive values were collected. The demographic clinical and laboratory details were tabulated in a Microsoft Excel (Redmond, Washington, US) spreadsheet. Statistical analysis was done via statistical package for the social sciences (SPSS) version 21 (IBM Corp., Armonk, NY, US), the Chi-Square test for demography, and Mann-Whitney test for laboratory parameters. A P-value of < 0.05 is considered significant. The cut-off values on Day 1 and peak values were derived from the receiver operating curve (ROC) with a confidence interval of 95%. Youden's index was used to determine the cut-off values. 

## Results

During the study period of one year, 3142 patients were managed in the hospital. Among them, 239 (7.6%) needed care in the ICU and they were included in the study. One hundred twenty-nine (54%) patients were shifted from the ward to the ICU due to deteriorating clinical conditions, and 110 (46%) of them required ICU care at the time of admission itself. The age of the patients ranged from 23 years to 97 years with a mean age of 62.5(±) years. A higher proportion of males (175) than females (65) in the ratio of 2.7:1 were provided ICU care. One hundred and fifty-four (64.5%) patients recovered from the disease, 83 (35.5%) died. Young patients (53.50±13 years) recovered well than the elderly (69±15 years, P-value = 0.00). No significant difference in gender was found between the groups (P = 0.789). Patients who recovered stayed for a shorter period (5±9 days) in the ICU than those who died (9±15 days, P = 0.041). The serial values of percentages of neutrophils, lymphocytes, platelet count NLR, and PLR are shown in Table 1.

**Table 1:** Dynamic changes (mean±standard deviation) of hematology parameters and comparison among survivors and non-survivors.

Characteristic	NEUTROPHIL 1 (%)	NEUTROPHIL 2 (%)	NEUTROPHIL 3 (%)	NEUTROPHIL 4 (%)	NEUTROPHIL 5 (%)	NEUTROPHIL 6 (%)	NEUTROPHIL 7 (%)	NEUTROPHIL 8 (%)
Survivors	81.55 (±16.8)	87.2 (±10.8)	87.8 (±9.2)	88.9 (±11.4)	89.5 (±9.3)	89.85 (±8.25)	90.3 (±8.5)	90.55 (±10.85)
Non-survivors	87.1 (±13.7)	91.55 (±5.5)	92.3 (±5.3)	92 (±7.4)	92.1 (±6.5)	93.3 (±5.6)	94.2 (±5.9)	93.7 (±6.6)
P-value	0.004	0.000	0.000	0.000	0.000	0.000	0.000	0.004
Characteristic	LYMPHOCYTES 1 (%)	LYMPHOCYTES 2 (%)	LYMPHOCYTES 3 (%)	LYMPHOCYTES 4 (%)	LYMPHOCYTES 5 (%)	LYMPHOCYTES 6 (%)	LYMPHOCYTES 7 (%)	LYMPHOCYTES 8 (%)
Survivors	10.35 (±12.7)	6.7 (±8.3)	6 (±6.1)	5.2 (±5.5)	5 (±5.55)	4.35 (±5.45)	4 (±5.1)	3.9 (±6.95)
Non-survivors	6.5 (±8.65)	4.65 (±3.6)	3.9 (±3.8)	3.3 (±3)	3.6 (±3.4)	3.2 (±3)	2.7 (±2.3)	2.6 (±1.7)
P-value	0.005	0.000	0.000	0.000	0.000	0.002	0.000	0.006
Characteristic	PLATELET 1 (lakhs/cu.mm)	PLATELET 2 (lakhs/cu.mm)	PLATELET 3 (lakhs/cu.mm)	PLATELET 4 (lakhs/cu.mm)	PLATELET 5 (lakhs/cu.mm)	PLATELET 6 (lakhs/cu.mm)	PLATELET 7 (lakhs/cu.mm)	PLATELET 8 (lakhs/cu.mm)
Survivors	2.18 (±1.3)	2.51 (±1.48)	2.7 (±1.45)	2.8 (±1.53)	2.83 (±1.8)	2.54 (±1.83)	2.51 (±1.86)	2.53 (±1.94)
Non-survivors	2.34 (±1.51)	2.56 (±1.66)	2.64 (±1.68)	2.73 (±2.4)	2.4 (±2.02)	2.41 (±2)	1.96 (±1.97)	1.99 (±1.6)
P-value	0.158	0.903	0.771	0.957	0.115	0.252	0.032	0.004
Characteristic	Neutrophil-lymphocyte ratio-1	Neutrophil-lymphocyte ratio-2	Neutrophil-lymphocyte ratio-3	Neutrophil-lmphocyte ratio-4	Neutrophil-lymphocyte ratio-5	Neutrophil-lymphocyte ratio-6	Neutrophil-lymphocyte ratio-7	Neutrophil-lymphocyte ratio-8
Survivors	7.96 (±11.45)	13.03 (±16.13)	14.77 (±18.77)	16.96 (±16.54)	17.87 (±19.4)	20.34 (±28.06)	22.52 (±25.41)	23.53 (±33.73)
Non-survivors	13.6 (±14.3)	19.06 (±25.55)	23.64 (±27.71)	28.36 (±32.76)	25.96 (±30.71)	28.88 (±37.14)	35.26 (±36.61)	35.72 (±25.15)
P-value	0.004	0.000	0.000	0.000	0.000	0.003	0.000	0.010
Characteristic	Platelet-lymphocyte ratio-1	Platelet-lymphocyte ratio-2	Platelet-lymphocyte ratio-3	Platelet-lymphocyte ratio-4	Platelet-lymphocyte ratio-5	Platelet-lymphocyte ratio-6	Platelet-lymphocyte ratio-7	Platelet-lymphocyte ratio-8
Survivors	0.18 (±0.39)	0.36 (±0.47)	0.45 (±0.57)	0.46 (±0.47)	0.48 (±0.39)	0.54 (±0.59)	0.59 (±0.59)	0.47 (±0.64)
Non-survivors	0.34 (±0.56)	0.66 (±0.71)	0.59 (±0.81)	0.79 (±0.96)	0.7 (±0.75)	0.75 (±0.79)	0.62 (±0.89)	0.51 (±0.82)
P-value	0.006	0.000	0.006	0.000	0.016	0.032	0.181	0.561

**Figure 1:**
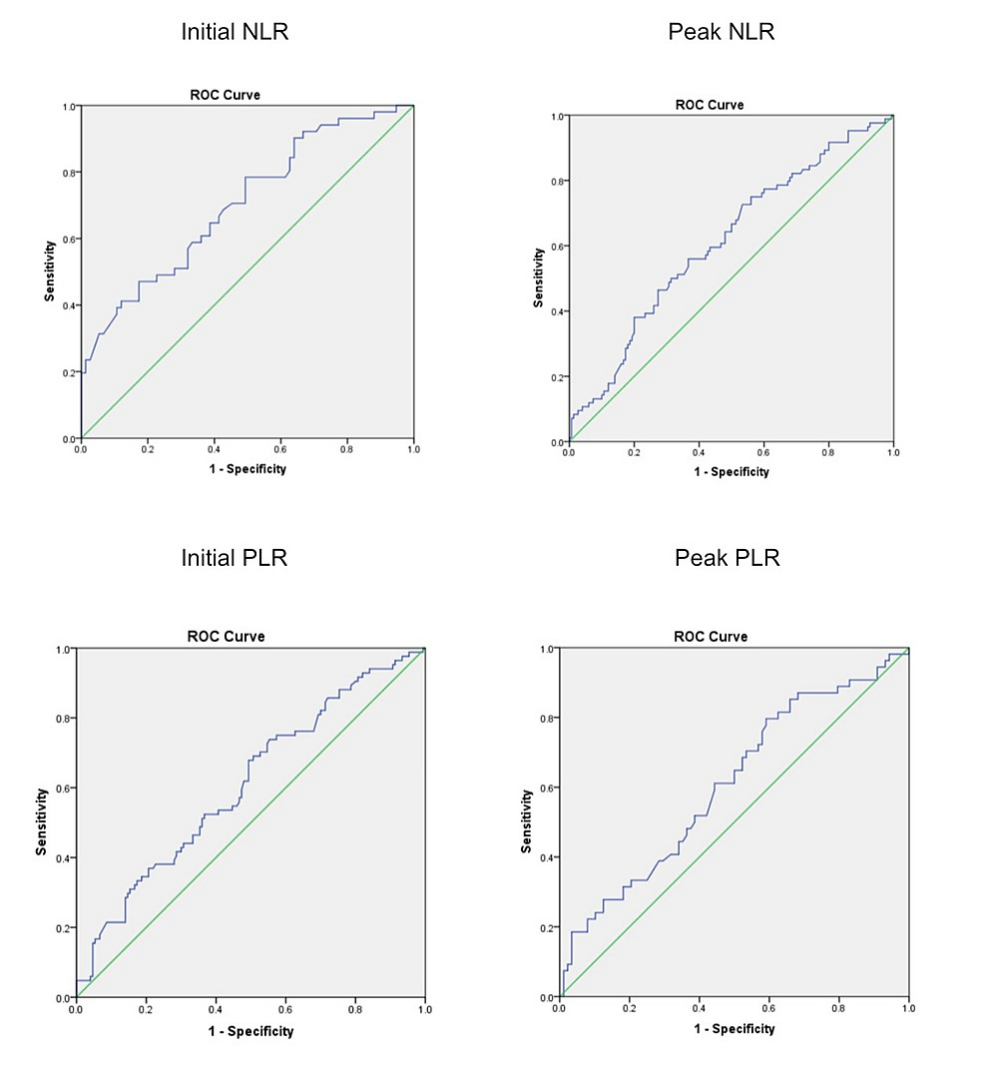
ROC curve analysis Figure 1 shows the ROC curve (C-statistics) for the neutrophil-lymphocyte ratio (NLR)  and platelet-lymphocyte ratio (PLR) on the day of admission at ICU (initial) and the peak value on any day.

There is a significant difference in neutrophil and lymphocyte percentage as well as the NLR, between the groups on all days. Though there is a downfall of platelet count, a significant fall was observed in the latter course in non-survivors. The PLR had been significantly high in the non-survivors. The cut-off values of NLR and PLR on the day of admission at ICU and the peak value on any da, are shown in Table 2. Figure 1 shows the ROC curve for the same.

**Table 2:** Area under the curve and critical values of hematology parameters.

Parameter	Cut-off value	Area under the curve (95% CI)	Sensitivity	Specificity
Initial Neutrophil	83.8	0.61 (0.54-0.69)	64.29%	56.67%
Peak Neutrophil	93	0.66(0.56-0.76)	62%	67.12%
Initial Neutrophil-lymphocyte ratio	6.7	0.61 (0.54-0.69)	56%	63.30%
Peak Neutrophil-lymphocyte ratio	40.95	0.70 (0.61-0.80)	64.30%	59.4%
Initial Platelet-lymphocyte ratio	160	0.61 (0.53-0.68)	67.90%	50.70%
Peak Platelet-lymphocyte ratio	400	0.61 (0.51-0.70)	61.50%	55.30%

## Discussion

Since December 2019, the healthcare system is working on the pathogenesis, diagnosis and management of COVID-19 disease. The utility of laboratory parameters in assessing severity and monitoring the course of the disease has been extensively worked up by clinical and laboratory personnel for the past one and a half years. Due to mutant variants as well as the impact of environmental and co-morbid conditions, heterogeneity in clinical presentations is being observed. The level of biomarkers may vary from population to population; hence research among different populations helps in assessing the severity and outcome in COVID-19. The inflammatory cytokine response in COVID-19 causes neutrophilia and this has been observed in many centres that manage patients with this disease. We observed a steady increase in the percentage of neutrophils in survivors and non-survivors but there is a statistically significant increase among the non-survivors. An initial neutrophil percentage above 80 needs ICU care and a higher value of 87% is associated with adverse outcomes. If it rises to 93%, mortality can be predicted. Lymphopenia has been consistently noted. This is linked to T-cell depletion. The downfall of lymphocytes has been observed in our experience. An initial lymphocyte percentage of 10% predicts adverse outcomes. Peak lymphocyte percentage of 5.2% is associated with mortality. Elevated NLR is associated with neutrophilia and lymphopenia. Studies done in China during the emergence of COVID-19 observed NLR between 3.18 and 6.29 in severe COVID-19 [3-6]. However, a single centre observation documented an NLR of 20.7 in severe cases [7]. Severity was also predicted by NLR >4.93 (Turkey), >8.78 (Pakistan) and >5 (Iran) [8-10]. Different single centre studies from India suggested NLR of >3.3 predict progression of the disease, >5.2 for admission to ICU and >5.59 in severe COVID-19 disease [11-13]. Mechanical ventilation was required when NLR was >4.6 [14]. We observed an initial NLR of 7.96 needed admission to ICU. The cut-off was 12.29 at admission, and a rising trend with 23.68 peak value in deceased persons predicted mortality. A study done at Wuhan, China observed an initial NLR of 7.13 [15] and peak NLR of 14.3, also associated with morbidity [16].

Table 3 shows NLR values among survivors and non-survivors and the critical values found in the present study and a few earlier ones. The higher cut-off in the study may be due to heterogeneity in time zone population, sample size, ethnicity and environmental conditions. Though we did observe thrombocytopenia in a few patients, the mean platelet value on all days was within range. But there had been a steady fall which was statistically significant in non-survivors in the latter stages. The PLR was significantly higher in non-survivors up to Day 6. But due to a fall in platelets as well as lymphocytes, PLR was non-significant in the latter course. An initial PLR of 160 is associated with an adverse outcome which is concomitant to the study by Yang et al. [7], and a peak PLR of 400 predicts mortality. Studies from other centres reveal a PLR of 180 (China and India), and 204 (India) [7,11,13]. The neutrophil and lymphocyte percentages were analysed in this study as many laboratories do not report absolute count, especially in resource-constrained areas. The limitation of the study is that co-morbid conditions were not attended to.

**Table 3:** Comparison of studies on NLR based on survival outcome NLR: Neutrophil-lymphocyte ratio

Study	Type	Survivor numbers	Survivor mean NLR ± SD	Non-survivor numbers	Non-survivor mean NLR ± SD	P-value	Critical values
Chennai, South India - One-year critical care retrospective study, 2020-2021	Initial	154	7.96±11.45	53	13.6±14.3	0.004	>12.29
	Peak		23.53±33.73		35.72±25.15	0.010	>23.68
Davangere, South India - Two-month retrospective study, 2020 ^[17]^	Initial	75	8.88±2.84	25	4.87±3.7	0.004	>4.7
Wuhan, China - 76 days, retrospective study, 2020 ^[16]^	Initial	297	2.53 (1.79-6.74)	52	15.96	<0.001	>7.13
	Peak		4.14 (2.11-12.32)		46.50	<0.001	>14.31
Wuhan, China - 46 days, retrospective study of older patients, 2020 ^[18]^	Initial	67	4.1±2.9	51	13.3±14.9	<0.001	>7.945
Wuhan, China, - 42 days, retrospective study of critically ill patients, 2020 ^[19]^	Initial	50	8.4±7.5	10	18.7±16.6	0.030	-
Wuhan, China - 27 days, retrospective study of adult patients, 2020 ^[20]^	Initial	268	3.40 (1.97-6.16)	47	12.27 (5.12-20.56)	0.001	>8.0

## Conclusions

Neutrophilia, lymphopenia, NLR, and PLR in patients at the time of admission for COVID-19 are cardinal laboratory findings that help in assessing the severity, early triaging, and continuous timely management. Dynamic monitoring of these biomarkers and their increasing trend are of prognostic potential. Also, NLR and PLR can be included in the software of the hematology analyzer to support the clinical team. The NLR of 6.7 and PLR of 160 require intensive care. The dynamic increase of NLR and PLR show worsening of the disease process and cut-offs at 40.95 and 400 predict mortality.
